# Utility of 3D multimodality imaging in the implantation of intracranial electrodes in epilepsy

**DOI:** 10.1111/epi.12924

**Published:** 2015-02-05

**Authors:** Mark Nowell, Roman Rodionov, Gergely Zombori, Rachel Sparks, Gavin Winston, Jane Kinghorn, Beate Diehl, Tim Wehner, Anna Miserocchi, Andrew W. McEvoy, Sebastien Ourselin, John Duncan

**Affiliations:** ^1^ Department of Clinical and Experimental EpilepsyUCL Institute of NeurologyLondonUnited Kingdom; ^2^ Department of NeurosurgeryNational Hospital for Neurology and NeurosurgeryLondonUnited Kingdom; ^3^ MRI UnitEpilepsy SocietyChalfont St PeterUnited Kingdom; ^4^ Centre of Medical Imaging and ComputingUCLLondonUnited Kingdom

**Keywords:** Epilepsy surgery, Image integration, Presurgical evaluation

## Abstract

**Objective:**

We present a single‐center prospective study, validating the use of 3D multimodality imaging (3DMMI) in patients undergoing intracranial electroencephalography (IC‐EEG).

**Methods:**

IC‐EEG implantation preparation entails first designing of the overall strategy of implantation (strategy) and second the precise details of implantation (planning). For each case, the multidisciplinary team made decisions on strategy and planning before the disclosure of multimodal brain imaging models. Any changes to decisions, following disclosure of the multimodal models, were recorded.

**Results:**

Disclosure of 3DMMI led to a change in strategy in 15 (34%) of 44 individuals. The changes included addition and subtraction of electrodes, addition of grids, and going directly to resection. For the detailed surgical planning, 3DMMI led to a change in 35 (81%) of 43 individuals. Twenty‐five (100%) of 25 patients undergoing stereo‐EEG (SEEG) underwent a change in electrode placement, with 158 (75%) of 212 electrode trajectories being altered.

**Significance:**

The use of 3DMMI makes substantial changes in clinical decision making.

Twenty percent to 40% of patients with focal epilepsy are refractory to medical management, and are candidates for epilepsy surgery.^1^ The minimum presurgical evaluation of these patients includes detailed history and examination, advanced epilepsy protocol magnetic resonance imaging (MRI) studies, video electroencephalography (EEG) telemetry, and neuropsychological and psychiatric assessment. Further neuroimaging studies such as fluorodeoxyglucose positron emission tomography (FDG‐PET), ictal single photon emission computed tomography (SPECT), functional MRI, diffusion tensor imaging (DTI) tractography, and magnetoencephalography (MEG) may provide further information to approximate the suspected epileptogenic zone, and relationship to eloquent cortical areas and white matter tracts.^2^

Intracranial EEG (IC‐EEG) is indicated in patients with medically intractable focal epilepsy when noninvasive investigations have failed to adequately define the epileptogenic zone.^3^ The decision to proceed to IC‐EEG, and the precise location and configuration of the implantation, arises from a multidisciplinary case review with all the noninvasive investigations. IC‐EEG may take the form of subdural grid electrodes, a combination of grid and depth electrodes, and by stereotactically placed depth electrodes (SEEG).

The use of integrated multimodality imaging in epilepsy surgery is well established.^4^, ^5^, ^6^ PET, fluid‐attenuated inversion recovery (FLAIR) MRI and SPECT can be coregistered and displayed on neuronavigation systems to unmask previously cryptogenic areas of interest to the operating surgeon and place them in an anatomic framework.^6^ Cortical motor, sensory, and speech representation mapped by functional MRI (fMRI), and corticospinal tracts derived from tractography, can also be displayed to enable resections of posterior temporal and extratemporal epileptogenic lesions in areas close to eloquent brain.^7^ Finally, electrode implantations can be visualized in the context of presurgical investigations by integration of postoperative CT reconstructions,^8^ and this is useful in the subsequent interpretation of IC‐EEG recordings.

There are no published data on the use of these tools in clinical practice. An online questionnaire distributed by our group (unpublished) indicated that three‐dimensional multimodality imaging (3DMMI) was felt to be useful in epilepsy surgery, and that it is underutilized in clinical practice. Eight of 40 respondents used 3DMMI in clinical practice. Twenty‐eight of 40 deemed 3DMMI as very useful and 7 of 40 deemed 3DMMI as slightly useful in the presurgical evaluation of epilepsy. The main barriers to implementation appeared to be cost (21/32) and the complexities of the process with a lack of local expertise (12/32).

We have previously reported on the feasibility of using 3DMMI to assist in the presurgical evaluation and surgical planning of epilepsy surgery in a busy tertiary center.^9^ The aim of this study is to validate the use of 3DMMI in clinical practice with a larger cohort, and demonstrate how it can be used throughout presurgical evaluation.

## Methods

### Ethics and recruitment

This project was approved by the joint research ethics committee of the National Hospital for Neurology and Neurosurgery (NHNN), and University College London (UCL) Institute of Neurology (ION). All participants were provided with patient information sheets and gave written, informed consent.

All individuals with medically refractory focal epilepsy, undergoing presurgical evaluation for placement of IC‐EEG and possible subsequent neocortical resection between August 2012 and August 2014, were invited to participate in the study.

### Image integration

Relevant structural and functional image acquisition was based entirely on clinical need, as determined by the patient's consultant neurologist, following discussion and consensus at the weekly multidisciplinary team meeting.

Patients undergoing IC‐EEG underwent a T_1_‐weighted Stealth MRI scan with gadolinium for the purposes of neuronavigation, and a 3D phase‐contrast MRI scan for the purposes of visualizing cortical surface vein anatomy. Individuals having SEEG also had a CT angiogram (CTA) to visualize intracerebral arteries.

All available structural and functional imaging modalities performed in the patient's presurgical evaluation were stored on a single workstation. See Tables S1–S3. Data pre‐processing was performed locally when required, prior to transfer to the workstation for the purpose of image integration.

Image integration was performed using the AMIRA (Version 5.4.0; Visualization Sciences Group, Boston, MA, US) software package, and more recently the EpiNav (CMIC, UCL, London, United Kingdom) software package. Image integration is a stepwise process by which each new modality is coregistered with the base anatomic image (Stealth T_1_‐weighted MRI with gadolinium injection), and display settings are adjusted using a range of tools to offer optimal data presentation and visualization.

The final multimodal image is a 3D‐volume‐rendered representation of the brain, with volume‐ or surface‐rendered clusters representing different modalities. The user can manipulate the final multimodal image in a number of ways, including alteration of the transparency of the brain and different modalities, rotation of the image in any plane, and changing the scaling of the image (Fig. S1). Modalities are color coded according to a fixed palette devised by our group (Table S2).

The data are presented at multidisciplinary team meetings, where the overall strategy of implantation (strategy) are decided, and at surgical meetings, where precise implantation details (planning) are decided. Once both the strategy and detailed planning are completed and approved by both neurophysiologist and operating neurosurgeon, the 3D models generated on AMIRA/EpiNav are exported onto the S7 StealthStation for intraoperative use during the implantation of IC‐EEG electrodes. The feasibility of this approach with AMIRA in routine clinical practice was demonstrated in a pilot study.^9^ Increasingly image integration and surgical planning has been performed on the EpiNav software in our center. EpiNav is an easy to use custom‐built software package dedicated to strategy and planning in epilepsy surgery. Like AMIRA, EpiNav can be used for rapid image integration and 3D visualization, In addition to this, EpiNav has added functionality, including a trajectory planner module for the placement of depth electrodes and a dedicated export module for archiving models and plans directly onto the S7 StealthStation (Table S3).

**Figure 1:**
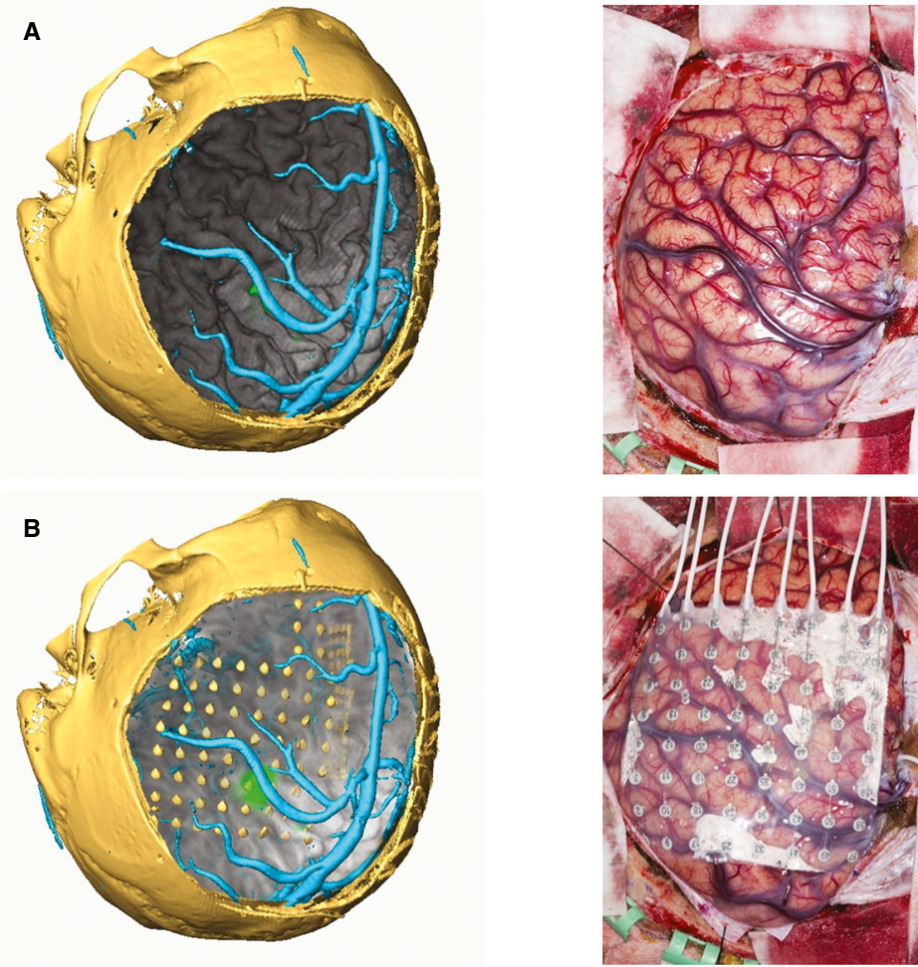
(**A**) Left: Volume rendering of cortex (gray) with addition of motor fMRI activation (green) and vein segmentation (cyan) in the context of the craniotomy bone flap, visualized by CT reconstruction. Right: Intraoperative photograph to show cortical surface and overlying vascular structures. (**B**) Left: Addition of subdural grids, with reconstruction on AMIRA. Right: Intraoperative photograph to show positioning of subdural grids on cortical surface.

Following implantation of electrodes, the localization of electrode contacts relative to structural and functional data can be examined by reconstructing the electrodes on the 3DMMI using AMIRA/EpiNav. Postimplantation CT imaging is coregistered to the T_1_ image (Fig. 1).

### Planning process

**Figure 2:**
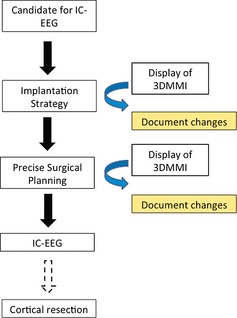
The workflow in this case series.

Two “milestone” discussions were identified in all patients undergoing consideration of IC‐EEG. The first is the implantation strategy and the second is the precise surgical planning (Fig. 2).

#### Implantation strategy

The implantation strategy is the definitive decision on whether to proceed to IC‐EEG, directly proceed to resective surgery, or abandon the possibility of surgical treatment. In the cases of IC‐EEG, a further decision is made on the favored strategy of using subdural grid electrodes, depth electrodes, or a combination of both, and how the desired coverage could be achieved. This meeting typically takes place a number of months prior to implantation. The implantation strategy is determined primarily by clinical neurophysiologists and neurologists in a case‐centered meeting. The presurgical evaluation, video telemetry, and neuroimaging are reviewed, followed by discussion led by the neurophysiologists on how best to proceed. Following consensus on the implantation strategy, the 3DMMI is disclosed to the group using the AMIRA workstation/Epinav workstation, and/or the S7 StealthStation. Any additional insights are recorded, as are any changes to the agreed strategy. The primary end point is whether disclosure of 3DMMI changes implantation strategy.

#### Precise surgical planning

The precise surgical planning specifies the implementation of the IC‐EEG. It is carried out by the consultant neurosurgeon and a neurosurgical trainee, and is performed on the EpiNav workstation and/or S7 StealthStation (AMIRA has no surgical planning functionality). This typically takes place one week before the implantation. The precise surgical planning consists of planning trajectories for the insertion of depth electrodes, the identification of cortical anatomy, and the planning of subdural grid placement. Following completion and documentation of the provisional plan, 3DMMI is disclosed on the EpiNav workstation/neuronavigation system, with the models displayed in 3D and also as contours on the orthogonal plane viewfinder. Any changes to the provisional plan are recorded. These may include changes in electrode arrangement, changes in electrode entry and target points, and changes to grid positioning. The primary end point is whether disclosure of 3DMMI changes the precise surgical planning.

## Results

### Demographics

Table 1 demonstrates the demographics and overall results of the 54 patients in the study.

**Table 1:** Demographics and surgical outcomes of the study population

Patient	Age/sex	Epilepsy duration (year)	Radiologic lesion	Description	Presumed EZ	Grid v depth	Software	Strategy change	Precise surgical planning change	Complications	Outcome	ILAE outcome (12/12)	Histology
1	45 F	30	Yes	L STG cavernoma, HS	L temporal	Grids	AMIRA	NR	Y	Nil	Cortical resection	3	HS
2	16 M	4	No	NA	L frontal	Grids	AMIRA	NR	N	Subdural hematoma	Cortical resection	3	NAD
3	31 M	18	Yes	FCD	R frontal	SEEG	AMIRA	NR	Y	Nil	Cortical resection	1	NAD
4	33 F	23	Yes	FCD	R parietal	Grids	AMIRA	NR	Y	Nil	Cortical resection	1 (4/12)	FCD type IIB
5	41 M	16	Yes	Heterotopia, HS	R temporal/parietal	SEEG	AMIRA	NR	Y	Nil	Cortical resection	3	HS
6	25 M	10	No	NA	L temporal	Grids	AMIRA	NR	Y	Nil	Cortical resection	1	NAD
7	27 M	17	No	NA	R parietooccipital	SEEG	EpiNav	NR	Y	Nil	Excluded		
8	19 F	10	Yes	Encephalomalacia	R occipital	SEEG	EpiNav	NR	Y	Nil	Declined further treatment		
9	43 M	29	Yes	FCD	R temporal	Grids	EpiNav	NR	Y	Nil	Awaiting resection		
10	25 M	19	Yes	FCD	L peri‐rolandic	Grids	AMIRA	N	Y	Nil	Excluded		
11	38 F	31	Yes	FCD	L frontal, insula	Grids	AMIRA	N	NA	Nil	Declined implantation		
12	45 F	30	Yes	DNET	R temporoparietal	Grids	AMIRA	N	Y	Nil	Cortical resection	1	DNET
13	60 F	35	Yes	FCD	L frontal	Grids	AMIRA	NR	Y	Nil	Cortical resection	1	FCD type IIB
14	21 M	15	Yes	FCD	R frontal	Grids	AMIRA	N	Y	Nil	Excluded		
15	27 F	24	E	FCD	L frontocentral	Grids	AMIRA	N	N	Infection	Cortical resection	1	FCD type IIA
16	39 M	36	Yes	Cavernoma	L superior parietal	Grids	AMIRA	N	Y	Nil	Cortical resection	4	Cavernoma
17	23 M	22	No	NA	L anterior frontal	Grids	AMIRA	N	N	Nil	Cortical resection	3	FCD type IIA
18	49 M	35	Yes	DNET	L frontal	Grids	AMIRA	Y	NR	Nil	Cortical resection	1	DNET
19	23 F	15	Yes	FCD	L frontal	Grids	AMIRA	N	N	Nil	Cortical resection	1	NAD
20	28 M	21	Yes	FCD	L parietal	Grids	AMIRA	Y	Y	Nil	Cortical resection	2	FCD type IIB
21	47 M	41	Yes	FCD	L frontal	Not done	AMIRA	Y	ND	Nil	Cortical resection	1	FCD type IIB
22	24 F	23	E	?FCD	R temporal, insula	SEEG	AMIRA	Y	Y	Nil	Cortical resection	1	HS
23	22 M	21	No	NA	L posterior quadrant	SEEG	AMIRA	N	Y	Nil	Cortical resection	1 (6/12)	FCD type IIA
24	52 M	19	Yes	HS	L frontotemporal	SEEG	AMIRA	Y	Y	Nil	Cortical resection	2 (4/12)	HS
25	29 M	11	Yes	Encephalomalacia	L frontotemporal	SEEG	EpiNav	Y	Y	Nil	Awaiting resection		
26	40 M	34	No	NA	L insula	SEEG	AMIRA	N	Y	Nil	Further implantation		
27	41 M	31	Yes	Encephalomalacia	R mesial frontal	SEEG	EpiNav	Y	Y	Nil	Awaiting resection		
28	20 M	8	Yes	Encephalomalacia	L centroparietal	Grids	EpiNav	N	N	Nil	Excluded		
29	54 M	46	Yes	FCD	L frontocentral	Grids	EpiNav	Y	N	Subdural hematoma	Cortical resection	1	FCD type IIB
30	18 M	7	E	?FCD	R temporal, insula	SEEG	EpiNav	N	Y	Nil	Cortical resection	1 (3/12)	HS
31	53 M	30	No	NA	R temporal	SEEG	EpiNav	Y	NR	Nil	Excluded		
32	30 F	24	Yes	Cortical tubers	R temporal	Grids	EpiNav	Y	N	Nil	Excluded		
33	39 M	10	No	NA	L mesial frontal	Grids	EpiNav	N	N	Infection	Cortical resection	4	NAD
34	44 M	21	Yes	LGG	Bitemporal	Grids	EpiNav	Y	ND	NA	NA		
35	55 F	20	No	NA	L temporal	SEEG	EpiNav	N	Y	Nil	Excluded		
36	47 M	8	No	NA	L parietal	Grids	EpiNav	Y	NR	Nil	Excluded		
37	40 M	35	No	NA	R occipital	SEEG	EpiNav	N	Y	Nil	Awaiting resection		
38	22 M	11	Yes	Encephalomalacia	L parietal	SEEG	EpiNav	N	Y	Nil	Awaiting resection		
39	32 M	15	Yes	DNET	R temporal	SEEG	EpiNav	N	Y	Nil	Awaiting resection		
40	19 M	14	No	NA	R frontal	SEEG	EpiNav	N	Y	Nil	Awaiting resection		
41	44 M	38	No	NA	R frontal	SEEG	EpiNav	N	Y	Nil	Awaiting resection		
42	37 F	17	Yes	DNET	R posterior quadrant	SEEG	EpiNav	Y	Y	Nil	Awaiting resection		
43	33 M	23	Yes	FCD	L frontal	Grids	EpiNav	N	Y	Infection	Excluded		
44	42 F	16	No	NA	R frontotemporal	SEEG	EpiNav	N	Y	Nil	Awaiting resection		
45	22 F	16	No	NA	R parietooccipital	SEEG	EpiNav	N	Y	Nil	Awaiting resection		
46	34 F	29	Yes	Encephalomalacia	L posterior temporal/insula	SEEG	EpiNav	N	Y	Hemorrhage	Excluded		
47	29 M	21	Yes	Schizencephaly, heterotopias	R temporal/parietal	SEEG	EpiNav	Y	Y	Nil	Awaiting resection		
48	29 F	7	Yes	FCD	Bitemporal	SEEG	EpiNav	N	Y	Nil	Excluded		
49	31 M	18	No	NA	L frontotemporal	SEEG	EpiNav	N	Y	Nil	Awaiting resection		
50	20 M	5	Yes	FCD	R frontal	SEEG	EpiNav	N	ND	NA	NA		
51	46 F	41	Yes	Cavernoma	R temporal	SEEG	EpiNav	N	ND	NA	NA		
52	37 M	4	Yes	LGG	R insula	SEEG	EpiNav	Y	NR	Nil	Awaiting resection		
53	29 M	14	No	NA	L frontal	SEEG	EpiNav	N	ND	NA	NA		
54	26 M	25	No	NA	R frontal	SEEG	EpiNav	N	NR	Nil	Excluded		

F, female; M, male; FCD, focal cortical dysplasia; HS, hippocampal sclerosis; LGG, low grade glioma; DNET, dysembryoplastic neuroepithelial tumor; NA, not applicable; E, equivocal; L, left; R, right; EZ, epileptogenic zone; SEEG, stereo‐electroencephalography; NR, not recorded; ND, not done; NAD, no abnormality detected.

The median age of the group was 32.5 years (range 19–60), and the median duration of epilepsy was 20.5 years (range 4–46). Thirty‐seven (69%) of 54 patients were considered to have epilepsy that originated outside the temporal lobe, and 21 (39%) of 54 patients had no clear structural lesion.

All patients had a model of their cortex built, derived from cortical segmentations of a T_1_‐weighted MRI generated by Freesurfer (Version 5.0.0; Martinos Center for Biomedical Imaging, Charlestown, MA, U.S.A.). These cortical models provided the anatomic framework on which to add other modalities.

A further 253 imaging models were created and added onto these cortex models, resulting in a mean 5.7 models per patient (Fig. S2). There were 82 models that helped to infer the epileptogenic zone, 52 models of tractography, and 69 models of venous/arterial vasculature. Four patients undergoing grid implantations had preoperative grid electrode models built. All 25 patients undergoing SEEG had models of individual electrode trajectories.

### Disclosure of 3DMMI

Fifty‐four patients were studied in this series, and the disclosure of 3DMMI changed some aspect of management in 43 (80%) of 54 cases. For each case, the change was in implantation strategy, precise surgical planning, or both.

#### Implantation strategy

**Figure 3:**
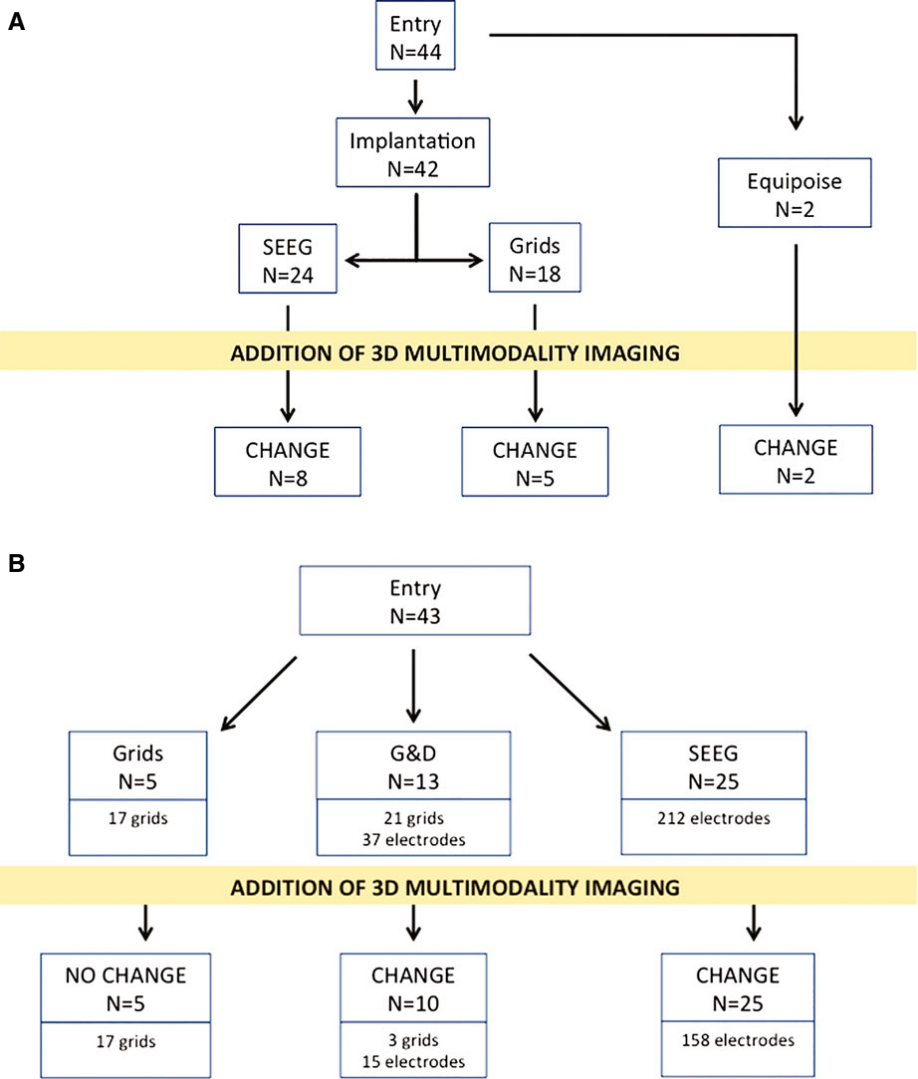
(**A**) An overview of the effect of 3DMMI on implantation strategy in this case series. (**B**) An overview of the effect of 3DMMI on precise surgical planning in this case series.

Forty‐four patients entered the implantation strategy arm. See Figure 3A for an overview of the effect of 3DMMI on implantation strategy. The remaining 10 patients in the study were not included in this arm of evaluating the impact of 3DMMI on implantation strategy.

In two patients there was clinical equipoise as to whether an implantation was necessary or resection could be recommended without IC‐EEG. With patient 21, this was due to the proximity of the presumed epileptogenic zone (EZ) to the motor cortex and corticospinal tract. Following disclosure of 3DMMI, the team felt it was safe to proceed with resection. With patient 52, it was thought not possible to completely resect an extensive structural lesion. Disclosure of the models led the team to consider that an implantation was necessary to precisely localize the EZ and offer safe resective surgery.

Forty‐two patients were initially put forward for an intracranial implantation: 18 for grid implantations and 24 for stereo‐EEG (SEEG) implantations. Disclosure of the models led to a change in the implantation strategy in 13 (31%) of 42 cases. There were 5 (28%) of 18 changes in grid strategy and 8 (33%) of 24 changes in SEEG strategy.

The details of the 15 changes in implantation strategy are listed in the Table 2.

**Table 2:** The changes in strategy following disclosure of 3DMMI in this case series

Patient	Initial strategy	Change in strategy	Reason
18	Grids	Addition of depth electrodes	Improve coverage around lesion
20	Grids	Addition of depth electrodes	Improve coverage around lesion
21	Equipoise	Proceed to resection	Good spatial corroboration between lesion and SPECT, anterior to motor areas
22	SEEG	Addition of depth electrodes	Target MEG dipole in insula
24	SEEG	Removal of depth electrodes	Difficult implementation of SEEG
25	SEEG	Re‐discussion in MDT	No initial consensus on agreed strategy
27	SEEG	Addition of depth electrodes	Improve coverage around lesion
29	Grids	Displacement of grid	Include coverage of PET hypometabolism
31	SEEG	Grids	Anterior frontal MEG dipole, more amenable to grid coverage
32	Grids	Addition of depth electrodes	Improve coverage of tubers
34	SEEG	Removal of depth electrodes	Improve efficiency of implantation
36	Grids	Displacement of grid	Include coverage of PET hypometabolism
42	SEEG	Removal of depth electrode	Improve efficiency of implantation
47	SEEG	Addition of depth electrode	Improve coverage to map optic radiation
52	Equipoise	Proceed to SEEG	Further localize EZ in large low‐grade glioma

MDT, multidisciplinary team meeting; SEEG, stereoelectroencephalography; SPECT, single photon emission computed tomography; MEG, magnetoencephalography; PET, positron emission tomography; EZ, epileptogenic zone.

#### Precise surgical planning

Forty‐three patients entered the precise surgical planning arm. Of the 11 patients not included in this arm, one patient declined to proceed with an implantation, one patient proceeded direct to a resection, four patients have not had precise surgical planning done and five patients did not have their planning recorded. See Figure 3B for an overview of the effect of 3DMMI on surgical planning.

Five patients were planned for grid implantations only, 13 patients were planned for a combination of grid and depth electrode implantations, and 25 patients were planned for SEEG implantations.

Disclosure of the models did not change the planning in the five cases using a strategy of subdural grids without depth electrodes. Disclosure of the models changed 10 (77%) of 13 cases using a combination of grids and depth electrodes. Disclosure of the models changed 25 (100%) of 25 cases using a strategy of SEEG. One hundred fifty‐eight (75%) of 212 electrodes were changed, with 124 changes to entry points, 28 changes to target points, and the addition of 6 further electrodes by the surgeons to optimize coverage. The most common reasons for changes to entry points were to minimize the risk of a vascular injury, by increasing the distance from a cortical surface vein (51) or by centering on the crown of the gyrus (44).

The details for the changes in SEEG implantation planning are listed in Table 3.

**Table 3:** The causes of change in precise planning of SEEG following disclosure of models

Changes in electrode	Number
Changes in entry point	124
Increase distance from vein	51
Center on gyral crown	44
Improve feasibility of trajectory	14
Use of gyral anatomy	8
Center on motor area (fMRI)	6
Avoid superficial temporal artery	1
Changes in target point	28
Target structural lesion	10
Increase distance from artery	8
Avoid electrode congestion	3
Improve feasibility of trajectory	3
Target PET/MEG	2
Target language areas (fMRI)	1
Target motor areas (fMRI)	1
Added electrodes	6
Total	158

### Outcomes

In total 46 patients in this series underwent an IC‐EEG (Table 1). Thirty‐five (76%) of 46 patients were put forward for a definitive cortical resection following identification of the presumed epileptogenic zone. Twenty‐one patients have had definitive cortical resections completed, and there is 1‐year follow‐up on 17 of these. Ten (59%) of 17 patients have International League Against Epilepsy (ILAE) Class I outcomes. Of the 11 patients excluded from surgery, the presumed epileptogenic zone was found at multiple sites in four cases, diffuse in three cases, overlapped with eloquent cortex in one case, and was not clearly identified in one case. In the remaining two cases, the patients did not have seizures and the implantations were abandoned after a given period of time.

## Discussion

### Summary

3DMMI was employed in the presurgical evaluation and surgical management of a consecutive series of patients with medically refractory epilepsy undergoing IC‐EEG implantation. Of the 54 patients studied, the disclosure of 3DMMI changed some aspect of management in 43 (80%) of 54 cases, with a 34% rate of change in strategy, and 81% rate of change in planning. The total added value of 3DMMI can be expressed in our series as follows: three patients moved out of clinical equipoise into resective surgery, IC‐EEG, and rediscussion at MDT, respectively; one patient underwent a change in IC‐EEG method, 11 patients underwent a fine‐tuning of implantation strategy; and 35 patients underwent a change in precise surgical planning, comprising of changes to three grids and 173 depth electrodes in total. This suggests that 3DMMI has a role to play in evaluation and management.

The implantation strategy, that is the design of the general method for implantation, changed in 15 of 44 individuals. These changes were not generally from one technique to another, rather a fine tuning of implantation design. This includes the addition of electrodes to improve coverage of structural, functional, and neurophysiologic data, and the reduction of electrodes to minimize surgical risk and increase efficiency.

The choice of technique for implantation is dependent on a number of factors, including hypothesis for EZ, need for functional mapping, surgical risk, and expertise of the treating team. In summary, it is our current view that, in general, patients with suspected seizure onset at the cortical surface, close to eloquent areas, will be more suited to subdural grid electrode implantation to facilitate extensive cortical mapping, whereas patients with suspected seizure onset at the depths of a sulcus, or areas of cortex not accessible by grids, are more likely to benefit from SEEG. The strategy for implantation was therefore based largely on the neurophysiologic hypothesis, which was arrived at prior to and independently of disclosure of 3DMMI. We would therefore not anticipate any substantial change in the chosen implantation technique with 3DMMI disclosure.

The results do, however, indicate how strategies can be improved when seen in the context of 3DMMI. This is in comparison to the previous convention in which some imaging is presented in 2D (i.e., structural MRI and FDG‐PET), and some imaging is not presented at all (fMRI, DTI tractography). With implantation of subdural electrodes it is possible to accurately gauge sizing of the grids and strips to ensure spatial coverage of underlying structural and functional areas of interest. We found this particularly useful in ensuring adequate cortical coverage for the purposes of functional mapping of the motor cortex and language areas, and in covering areas of interest such as FDG‐PET hypometabolism. With SEEG, the 3D spatial arrangement of depth electrodes is difficult to appreciate and communicate in 2D form. Adjustments can be made to “fill” any obvious gaps in spatial coverage, and to remove electrodes that duplicate coverage and cause congestion. Furthermore, deep targeting of functional areas of interest is only possible with 3DMMI. We conclude that designing implantations is suboptimal without the ability to visualize the 3D spatial relationships between regions of interest and implanted contacts.

Precise surgical planning, that is, the planning of the details of the implantation, changed in 35 of 43 cases.

Disclosure of 3DMMI did not change surgical planning in cases of grid implantation. Grid planning is essentially the determination of the sizing and positioning, which is completed in the strategy phase. Neurosurgeons use 3DMMI to inform the correct placement of the grids, and the optimal placement of the overlying craniotomy, but there is little discrete planning to record.

Disclosure of 3DMMI changed surgical planning in all 25 cases of SEEG. In total, the majority of electrodes (158/212, 75%) were altered following disclosure of 3DMMI. This supports the notion that 3DMMI is helpful for the design and planning of SEEG. Entry points were changed primarily to reduce the risk of hemorrhagic complications associated with cortical vein injury, and to center on the gyral crown to avoid encroaching pial boundaries (see Fig. S3). Other reasons for change included accurate targeting of superficial cortical structures such as motor cortex, and the facilitation of improved trajectory angles through the skull that were easier to implement. Target points were changed primarily to locate important structural and functional areas of interest, such as deep areas of focal cortical dysplasia or FDG‐PET hypometabolism.

After planning all of the individual electrodes, 3DMMI allowed the clinical team to review the assembled implantation, and identify defects in spatial coverage and areas of electrode congestion. In some cases this led to the addition of depth electrodes, or the further alteration in individual depth electrode trajectory.

In practice, surgical planning of SEEG is a complex process; in any electrode arrangement, the entry and target point of a solitary electrode can affect the entire arrangement, and it is not uncommon to make several changes following disclosure of 3DMMI. We concede that it is possible to plan in 2D and arrive at reasonable electrode configurations with safe trajectories. However, 3D representations of gyral anatomy and vasculature, in particular, allow the surgeon to make small changes to electrode positioning with a clear understanding of how this affects proximity to other structures. We believe this is the crucial difference to planning in 2D, and makes for a more informed method that optimizes safety considerations.

### General

Throughout this study, there has been a development in understanding how 3DMMI can be used to improve clinical care. In terms of modalities, structural and functional data appear most relevant in determination of strategy, whereas vascular and gyral anatomy appear most relevant in precise planning. However, disclosure of 3DMMI does not always represent a change in decision making, but rather an added tool to support and inform decision making. Feedback from the neurophysiology and neurosurgery teams suggests the following benefits of 3DMMI in clinical practice.

1. 3D spatial concordance between structural and functional localizing investigations for epileptogenic zone.
2. 3D spatial relationship of presumed epileptogenic zone to surrounding critical structures.
3. Avoidance of hemorrhagic complications in the planning and implementation of SEEG.
4. Planning of grid placement for purposes of functional cortical mapping.
5. Use of vessel segmentation and models of segmented gyral anatomy to provide navigational tool in craniotomy.

With subdural grid implantations and subsequent cortical resections, 3DMMI can be used by the surgeon as an additional navigation tool. For example, models of cortical veins, allied with segmentations of complex gyral anatomy such as the central sulcus and pre‐ and postcentral gyrus, can provide valuable corroboration tools to orientate the surgeon^10^ and to check neuronavigation registration accuracy. This becomes particularly powerful when the models are propagated as 2D models through the operating microscope.

In addition to using 3DMMI in presurgical evaluation, we also used 3DMMI postoperatively to demonstrate the spatial positioning of electrode contacts using the methodology outlined by Chamoun et al.^8^ (see Fig. 1B). This involves the coregistration of postoperative CT to the preoperative imaging, followed by surface extraction and thresholding to build 3D models of implanted electrodes (Fig. 1). This provided a crucial tool in the understanding of implantation accuracy and outcome, and interpreting the generated neurophysiologic data. This is especially the case in those grid electrode implantations where it is not possible to directly visualize and photograph the cortical surface, for example, orbitofrontal cortex, and in depth electrodes targeting deep structures, for example, anterior hippocampus. There is the obvious limitation of brain shift, which is more pertinent to grid electrode implantations with associated large craniotomies, but can also occur in SEEG with the escape of cerebrospinal fluid and effect of gravity on head position. This can be overcome by acquiring postoperative MRI, which directly shows electrode positioning in an anatomic context. However, individual centers have to pass their own safety testing prior to acquisition of MRI with implantations in situ. In addition, there is the possibility of electrode contact shift during the recording period and after postoperative imaging, which may falsely localize the epileptogenic zone on the cortical surface.

### Implications

This study demonstrates the potential role of 3DMMI in clinical practice. We anticipate that 3DMMI will become increasingly important in the future, for several reasons. Ongoing research in advanced imaging techniques, such as voxel‐based analysis, EEG‐fMRI, and magnetoencephalography,^2^ will add further modalities to be considered in 3D anatomic space and presented to the clinical team. At the same time, there is a trend toward less‐invasive and better‐tolerated investigations and treatments in neurosurgery, with much hope for neuroablative techniques such as SEEG‐guided radiofrequency thermocoagulation, MR‐guided laser therapy, and ultrasound therapy.^11^ A planning system that presents 3DMMI multimodal data and communicates with existing neuronavigation software will be essential in the delivery of future implantations and treatments.

## Limitations

The aim of the current study was to describe how the use of 3DMMI can change clinical management. The study does not specifically address whether the changes in practice, as a direct result of disclosure of the 3DMMI, make any substantial difference to the outcomes in these patients. Although it is intuitive to think that more data will lead to better decision making and therefore better outcomes, we do not have evidence for this.

Class I evidence for the use of 3DMMI in epilepsy surgery would require the design of a randomized controlled trial. This is problematic in clinical practice for the following reasons.

1. Epilepsy, even if restricted to refractory focal epilepsy, is an extremely heterogeneous condition with a heterogeneous population group.
2. Epilepsy surgery is relatively uncommon, with low numbers.
3. Assessing outcome after epilepsy surgery is complex.
4. Use of models already partially integrated into the clinical pathway.

An alternative approach is to examine secondary end points that are surrogate markers for outcome. First, did the patients undergoing IC‐EEG achieve a satisfactory conclusion to their implantation with regard to seizure localization? Thirty‐five of 46 patients have been put forward for cortical resection and 11 of 46 patients have been excluded from resective surgery in this series. The epileptogenic zone was satisfactorily identified in up to 43 of the 46 cases studied. This indicates that the final implantations were well designed. It will be some time before robust seizure outcome data are available in this patient group. At the present time 10 (59%) of 17 patients have ILAE Class I outcomes at 1 year of follow‐up. These results are consistent with the literature when one considers the difficult patient group, with high rates of nonlesional and extratemporal epilepsy.

Second, did the patients undergoing IC‐EEG have any complications as a result of IC‐EEG implementation? There was one case of a hemorrhagic complication using a frameless stereotactic method for SEEG implantation of a total of 212 electrodes implanted (0.5%). This patient was asymptomatic and did not require any further treatment. With the grid cases, there were two cases of subdural hematomas that required evacuation and precluded continued IC‐EEG recording, and two cases of infection. This complication rate is comparable with that in the literature.^12^ Overall these events were not thought to be a result of the use of 3DMMI, and these surrogate markers are suggestive of good clinical practice.

A further caution is that the use of 3DMMI is dependent on the generation of high‐quality input data, which is well understood by the end user. We know that some data sets will be reliable and reproducible such as structural MRI, and other sets will have considerable interuser variability such as tractography.^13^ Interpretation of these within an integrated data set requires differential levels of caution and confidence by the neurophysiologist and neurosurgeon. Taking the example of tractography, we recommend using this as a guide to the orientation of large white matter tracts, and intraoperative mapping is required for definitive identification. Similarly, the sensitivity of 3D phase contrast MRI is limited, and in our experience the segmentation of cortical veins derived from this modality is incomplete at the convexity of the hemispheres. It is therefore crucial for the surgeon to check the “probes eye view” planes on the MRI with gadolinium, prior to implementing a trajectory planned with the vascular models.

The complexity of presurgical evaluation gives rise to two further limitations with this study. First, there is the difficulty associated with capturing benefits. The terms “strategy” and “planning” are divisions made in a stepwise process. Presurgical evaluation extends over many months and often involves the exchange of ideas between the treating neurology and surgical team at multiple points along the course. To capture the impact of 3DMMI, a degree of rigidity was applied to this process, with the distinction of implantation strategy from precise surgical planning.

Second, there is difficulty capturing the entire workflow for a given number of patients. Because this study ran for 2 years, it represents a snapshot of the presurgical evaluation and surgical management in our unit. Although 54 patients passed through the study, only 44 were evaluated for strategy, 43 for precise surgical planning and 46 for outcome. The reasons for this were given earlier and reflected in the chronologic order of Table 1; any attempt to homogenize the results to only patients passing through the entire process with 1‐year follow‐up would result in much data loss.

Finally, the generalizability of our findings needs to be confirmed in other centers that do not have the same degree of interest and expertise in 3DMMI. The EpiNav software is being made available to other units for this express purpose.

## Further Work

The next logical step to this work is to examine the utility of 3DMMI in planning definitive cortical resections. This is especially pertinent following SEEG implantations, where the EZ may be deep, difficult to access, and without clear anatomic borders. In the present study for extratemporal resections, the surgeon used the spatial positioning of implanted electrodes implicated in seizure origin, and aimed to remove the entirety of any segmented structural lesion present. In the future, we plan to generate resection models to guide surgery that incorporate advanced imaging techniques and neurophysiologic data and that respect anatomic boundaries.

## Disclosure of Conflict of Interest

John Duncan has received institutional grant support from Eisai, UCB Pharma, GlaxoSmithKline, Janssen Cilag, Medtronic, and GE Healthcare. Andrew McEvoy has received support from UCB, Baxter, and Cyberonics The remaining authors have no conflicts of interest. This publication presents independent research supported by the Health Innovation Challenge Fund (HICF‐T4‐275, Programme Grant 97914), a parallel funding partnership between the Department of Health and Wellcome Trust. The views expressed in this publication are those of the author(s) and not necessarily those of the Department of Health or Wellcome Trust. We confirm that we have read the Journal's position on issues involved in ethical publication and affirm that this report is consistent with those guidelines.

## Supporting information


**Table S1.** The imaging modalities used in this series. ES, Epilepsy Society; NHNN, National Hospital for Neurology and Neurosurgery; MEG, magnetoencephalography; SPECT, single photon emission computed tomography; FDG‐PET‐ fluorodeoxyglucose positron emission tomography; DTI, diffusion tensor imaging.
**Table S2.** The color palette used to display multimodality in this series.
**Table S3.** Comparison of AMIRA and EpiNav software.

**Figure S1.** Volume rendering of cortex (gray) displayed in AMIRA with the following associated modalities: focal cortical dysplasia (red), FDG‐PET hypometabolism (purple), hand motor fMRI (green), corticospinal tractography (blue), veins (cyan).

**Figure S2.** The imaging modalities used in the case series.

**Figure S3.** 3D model of the precise surgical planning of SEEG on EpiNav. (**A**) Prior to disclosure of 3DMMI; (**B**) following disclosure of 3DMMI.
