# Supplementary material for: Utility of 3D multimodality imaging in the implantation of intracranial electrodes in epilepsy
Source: Epilepsia. 2015 Feb 5;56(3):403–13. doi: 10.1111/epi.12924 (PMC4737214; doi:10.1111/epi.12924)
Supplement: Supplementary file 1 — Table S1. The imaging modalities used in this series. ES, Epilepsy Society; NHNN, National Hospital for Neurology and Neurosurgery; MEG, magnetoencephalography; SPECT, single photon emission computed tomography; FDG‐PET‐ fluorodeoxyglucose positron emission tomography; DTI, diffusion tensor imaging. Table S2. The color palette used to display multimodality in this series. Table S3. Comparison of AMIRA and EpiNav software. [file EPI-56-403-s001.docx]

| **Modality** | **Location** | **Scanner** | **Field of view (APxRLxIS)** | **Voxel size (APxRLxIS)**  **(mm)** |
| --- | --- | --- | --- | --- |
| 3D T1 FSPGR | ES | GE 3T Signa HDx | 256x256x166 | 0.94x0.94x1.1 |
| Coronal T2 FLAIR | ES | GE 3T Signa HDx | 256x160x32 | 0.94x1.5x3.5 |
| Navigation T1 with gadolinium | NHNN | Siemens Avanto 1.5T | 512x512x144 | 0.5x0.5x1.5 |
| MRI 3D phase contrast | NHNN | Siemens Avanto 1.5T | 256x256x160 | 0.85x0.85x1 |
| CT angiogram | NHNN | Siemens Somatom Definition AS | 512x512x383 | 0.43x0.43x.75 |
| MEG dipole | NHNN |  |  |  |
| Ictal-interictal SPECT | UCH | GE Discovery 670 | 128x128x49 | 3.9x3.9x3.9 |
| FDG PET | UCLH | GE DST/DVCT | 128x128x47 | 1.95x1.95x3.3 |
| DTI | ES | GE 3T Signa HDx | 128x128x60 | 1.88x1.88x2.4 |
| Functional MRI | ES | GE 3T Signa HDx | 128x128x58 | 1.87x1.87x2.5 |

Supplementary Table 1. A Table to show the Imaging Modalities used in this series (ES-Epilepsy Society, NHNN-National Hospital for Neurology and Neurosurgery, UCLH- University College London Hospital, FSPGR-FastSpoiledGradientRecalledEcho, MEG-magnetoencephalography, SPECT-single photon emission computed tomography, FDG PET- fluorodeoxyglucose positron emission tomography, DTI-diffusion tensor imaging, AP- anterior posterior, RL – right left, IS – inferior superior)

| Region of interest | Red component | Green component | Blue component | Overall | Colour bar |
| --- | --- | --- | --- | --- | --- |
| Lesion | 237 | 17 | 45 | Red |  |
| MEG | 203 | 23 | 207 | Violet |  |
| Language | 244 | 126 | 28 | Orange |  |
| Motor area | 0 | 255 | 0 | Light green |  |
| Sensory area | 0 | 128 | 0 | Dark green |  |
| Veins | 0 | 255 | 255 | Cyan |  |
| Arteries | 186 | 44 | 44 | Dark red |  |
| White matter tracts | 17 | 54 | 239 | Blue |  |
| EEG-fMRI | 113 | 61 | 55 | Brown |  |
| PET hypometabolism | 130 | 0 | 130 | Purple |  |
| SPECT hyperperfusion | 255 | 185 | 255 | Pink |  |
| IC-electrodes | 255 | 255 | 0 | Yellow |  |
| Skull | 224 | 224 | 224 | White |  |

Supplementary Table 2 A Table to show the RGB colour palette for display of multimodality imaging in this series

|  | AMIRA | EpiNav^TM^ |
| --- | --- | --- |
| Availability | Commercial software, license required | In-house software, In development |
| Technical skills needed | Medium | Low |
| User friendliness | Medium | Medium |
| Duration of processing* | 2-3 hours | 1-2 hours |
| Visualisation | Volume-rendering | Surface-rendering |
| Vessel extraction | Multi-step | Single step |
| Planning | Not available | Trajectory planning module |
| Export to S7 Stealthstation | Difficult | Easy |
| Post-operative electrode reconstructions | Easy | Easy |

- Not inclusive of data collation and pre-processing

Supplementary Table 3. A Table to show a comparison of AMIRA and EpiNav software packages
