# Supplementary figures and images for: Utility of 3D multimodality imaging in the implantation of intracranial electrodes in epilepsy
Source: Epilepsia. 2015 Feb 5;56(3):403–13. doi: 10.1111/epi.12924 (PMC4737214; doi:10.1111/epi.12924)

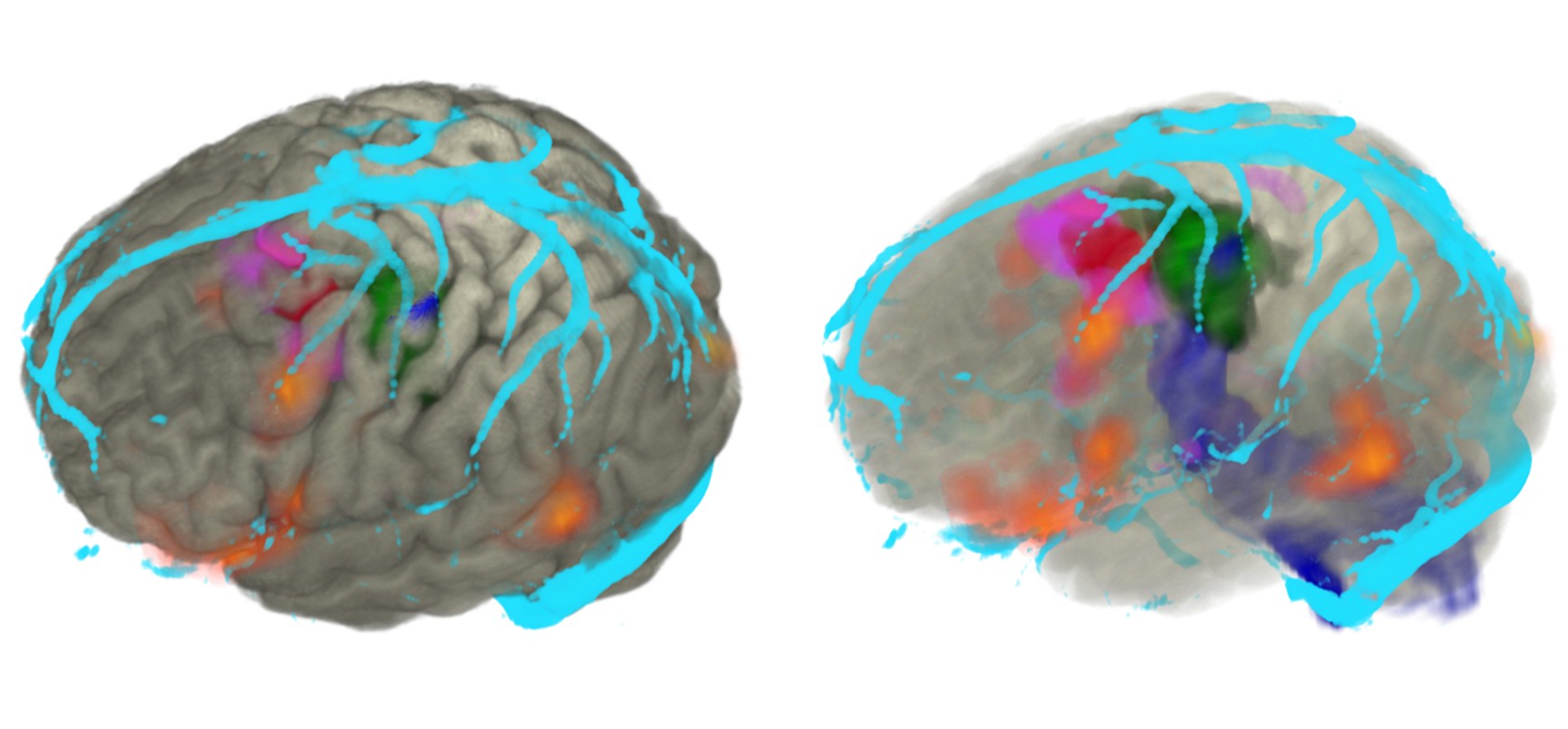

Supplement: Supplementary file 2 — Figure S1. Volume rendering of cortex (gray) displayed in AMIRA with the following associated modalities: focal cortical dysplasia (red), FDG‐PET hypometabolism (purple), hand motor fMRI (green), corticospinal tractography (blue), veins (cyan). [file EPI-56-403-s002.tif]

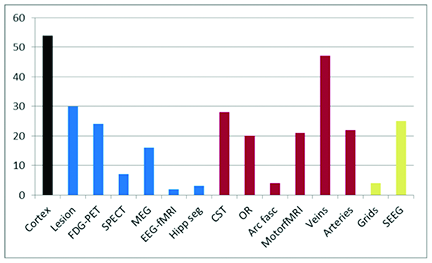

Supplement: Supplementary file 3 — Figure S2. The imaging modalities used in the case series. [file EPI-56-403-s003.tif]

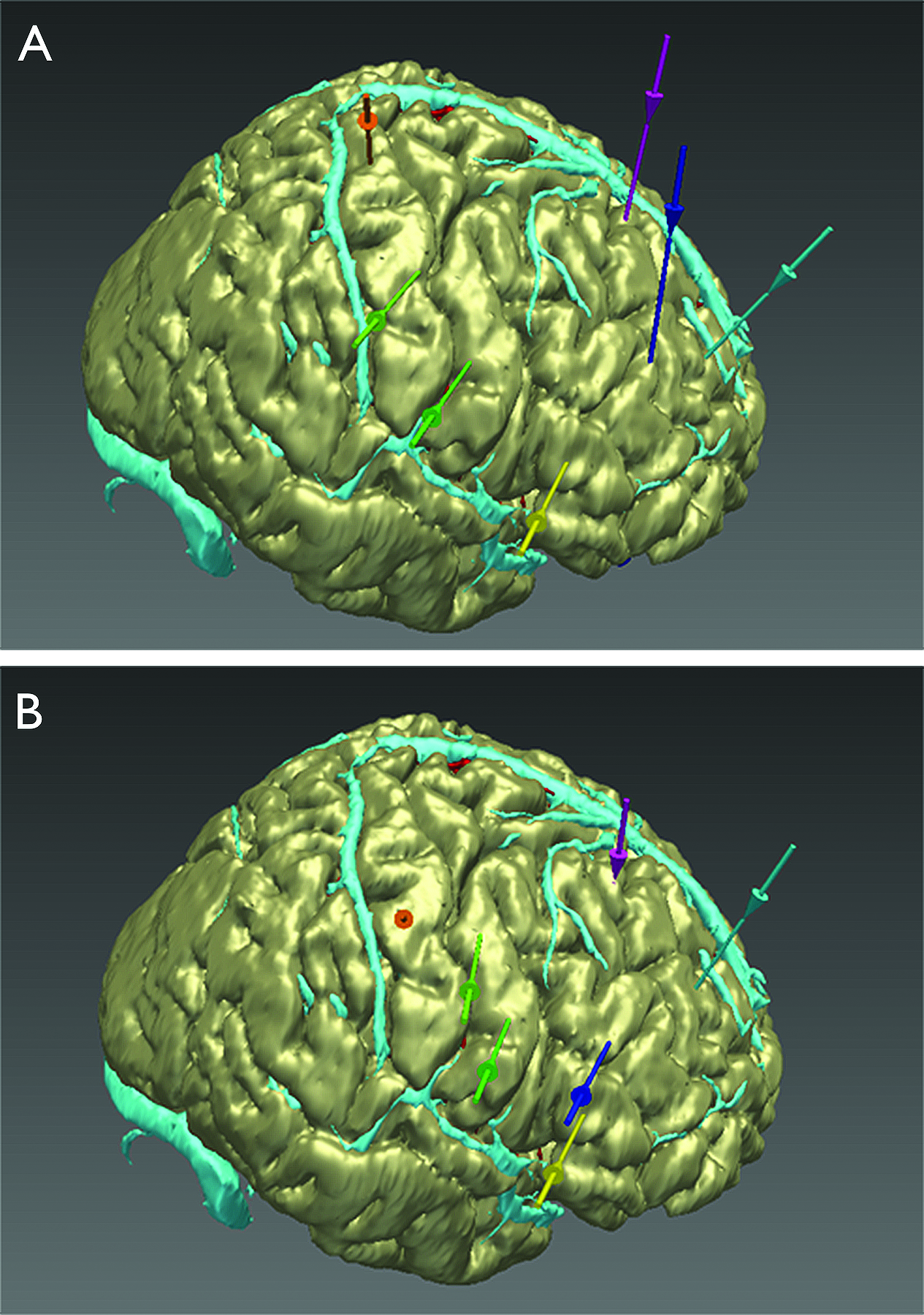

Supplement: Supplementary file 4 — Figure S3. 3D model of the precise surgical planning of SEEG on EpiNav. (A) Prior to disclosure of 3DMMI; (B) following disclosure of 3DMMI. [file EPI-56-403-s004.tif]
